# Effects on blood parameters from hand-arm vibrations exposure

**DOI:** 10.1177/07482337231173733

**Published:** 2023-04-28

**Authors:** Niclas Johansson, Oscar Ragnebro, Albin Stjernbrandt, Pål Graff, Ing-Liss Bryngelsson, Per Vihlborg

**Affiliations:** 1 Department of Laboratory Medicine, Faculty of Medicine and Health, 570872 Örebro University Hospital, Örebro, Sweden; 2 Department of Occupational and Environmental Medicine, Faculty of Medicine and Health, 596174 Örebro University, Örebro, Sweden; 3 Section of Sustainable Health, Department of Public Health and Clinical Medicine, 59588 Umeå University, Umeå, Sweden; 4 National Institute of Occupational Health, 70672 STAMI, Oslo, Norway; 5 Department of Geriatrics, Faculty of Medicine and Health, 596174 Örebro University, Örebro, Sweden

**Keywords:** Hand-arm vibration, vibration white fingers, Raynaud’s syndrome, vibration exposure, neutrophilic granulocytes, blood viscosity

## Abstract

Vibration exposure from handheld tools can affect the hands with neurological symptoms and vibration-induced Raynaud’s phenomenon (VRP). The underlying pathophysiological mechanisms are not fully known, however, changes in the composition of blood parameters may contribute to VRP with an increase in blood viscosity and inflammatory response. The aim of this study was to examine the effect on blood parameters in capillary blood from fingers that had been exposed to a vibrating hand-held tool. This study involved nine healthy participants who had been exposed to vibration and an unexposed control group of six participants. Capillary blood samples were collected before and after vibration exposure for the exposed group, and repeated samples also from the control group. The exposed groups were exposed to vibration for a 15-min period or until they reached a 5.0 m/s^2^ vibration dose. Analysis of blood status and differential counting of leucocytes was performed on the capillary blood samples. The results of the blood samples showed an increase in mean value for erythrocyte volume fraction (EVF), hemoglobin, red blood cell count, white blood cell count and neutrophils, as well as a decrease of mean cell volume, mean cell hemoglobin, and mean cell hemoglobin concentration. The increase of EVF and neutrophils was statistically significant for samples taken from the index finger but not the little finger. Even though the study was small it showed that an acute vibration exposure to the hands might increase EVF and neutrophilic granulocytes levels in the capillary blood taken from index fingers.

## Introduction

Exposure to hand-arm vibration (HAV) from use of hand-held vibrating tools is common in Sweden. According to Statistics Sweden, approximately 10% of all employed people in Sweden are exposed to vibration from handheld tools for at least a quarter of their working day (Klevestedt., 2018). Exposure to HAV can induce hand-arm vibration syndrome (HAVS) (Nilsson et al., 2017). HAVS includes three types of symptoms: vascular, neurosensory, and musculoskeletal. The vascular symptoms, often referred to as secondary Raynaud’s phenomenon or vibration-induced Raynaud’s phenomenon (VRP) are associated with vibration exposure for an extended period of time (Stoyneva et al., 2003).

The pathophysiological mechanisms underlying VRP are not fully known, though it is accepted that nerve damage can occur during vibration exposure (Nilsson et al., 2017). HAV causes mechanical damage that can affect the function of blood vessels and nerves (Gemne, 1994; Prete et al., 2014).

It is considered likely that mechanical damage can cause a combination of dysfunction of nerves, local vascular regulation, and changes in blood parameters, which contribute to vasospasm (Stoyneva et al., 2003).

Autonomous imbalance has been observed, showing either increased sympathetic activity or reduced parasympathetic response within HAVS-diagnosed patients, which can lead to increased vasoconstriction (Heinonen et al., 1987; Bovenzi, 1986). The increase in peripheral resistance in fingers can also be explained by a difference in the affinity of ligands to local receptors that effects vasodilation or constriction. Further, mechanical damage that causes nerve damage, specifically when the damage occurs peripherally in fingers that contain vasodilating neuropeptides, might explain the increased resistance in blood vessels (Stoyneva et al., 2003). An increased level of endothelin-1 has been found in patients with HAVS when exposed to cold (Eriksson et al., 2020). The increased level of endothelin-1 attenuates the relaxation effect of smooth muscles in the vessels that is usually regulated by NO, which can also be affected by vibration exposure (Noël, 2000). Endothelin-1 also has a modulating effect on the release of neurotransmitters from sympathetic nerve ends, thereby causing multiple effects to increase tonus (Damon, 1998).

An increase in blood viscosity has been observed among patients diagnosed with VRP. This hyper-viscosity, together with other mechanisms, could contribute to the lack of oxygen in the tissue (Okada et al., 1987). Vibration exposure also increases thrombocyte aggregation, and thereby the blood viscosity (Kent et al., 1994).

Leucocytes normally have difficulties passing through small capillaries. The blood flow in the capillaries can be further reduced due to activated leucocytes forming aggregating structures with each other or adhering to the vessel walls (Stoyneva et al., 2003; Nawaz et al., 2022). VRP patients have been shown to have an increased production of the pro-inflammatory substance leukotriene B4, which has an aggregating effect on leucocytes that can contribute to decreased oxygenation (Lau et al., 1992).

Earlier studies have shown changes in erythrocyte volume fraction (EVF) and plasma viscosity after vibration exposure. In these studies, venous blood was analyzed and it was shown that there was no difference between subjects with VRP and a control group, though a significant difference was observed before and after exposure (Greenstein and Kester, 1997).

The aim of this study was to investigate the impact of a vibrating handheld tool on blood parameters in capillary blood from fingers. To achieve this, the blood status and leucocyte differential count were analysed before and after exposure to vibration.

## Methods

The participants of this intervention study were all office workers without any previous occupational hand-arm vibration exposure. They were informed and gave their consent to participate in this study. To qualify for inclusion, study participants could not have any neurological or rheumatic disease.

Before exposure to hand-arm vibration, capillary blood was collected from the center of the index finger and the tip of the little finger in the dominant hand. Only the index finger was used for sampling in the control group. Blood was collected using a Safety-Lancet Super manufactured by SARSTEDT AG & Co. KG (Nümbrecht, Germany), and stored in 0.5 mL Microcollect tubes (K2E K2EDTA) Greiner Bio-One GmbH (Kremsmünster, Austria).

The participants were exposed to vibration by using a handheld polishing tool, CP240B from Best Tools USA (Gravette, USA), for a 15-min period or until they reached the Swedish limit value of 5.0 m/s^2^ calculated as cumulative daily exposure A(8) according to standard ISO 5349-2:2001 (International Organization for Standardization, 2001). The vibration exposure dose was measured by an SV 150 hand-arm triaxial accelerometer manufactured by SVANTEK SP.Z O.O (Warszawa, Poland), and the sensors were placed on both handles of the polishing tool.

After vibration exposure, capillary blood samples were collected again. To avoid sampling in the same location as before exposure, samples were taken on the opposite side of the fingertip. Also, an unexposed control group of six participants were included, in which blood was collected in the same procedure.

The samples were directly sent to a laboratory for analysis of blood status and a five-part differential count of leucocytes. The analyses were performed by the Laboratory Medicine Department of the University Hospital, Örebro, Sweden.

The following hematological parameters were measured: erythrocyte volume fraction/hematocrit (EVF); hemoglobin (Hb); red blood cell (RBC) count; white bloodcell (WBC) count; platelet/thrombocyte (PLT) count; mean cell volume (ercMCV); mean cell hemoglobin (ercMCH); and mean cell hemoglobin concentration (ercMCHC). WBC were differentially counted and determined in absolute values of lymphocytes, neutrophils, eosinophils, basophils, and monocytes for each participant.

Descriptive statistics were used to assess baseline characteristics of the population.

Because of the size of the study population, the Wilcoxon signed-ranks test was used to determine statistical significance, as this test is a non-parametric test. IBM SPSS Statistics 25 was used for the statistical analysis.

### Ethical considerations

Capillary sampling can cause discomfort but is not associated with any risk. The vibration exposure was below the legislated threshold of the Swedish Work Environment Authority. The Swedish Ethical Review Authority (DNR 2019-03883) approved the study protocol.

## Results

Six men and three women were in the exposed group. Three men/women were in the control group.  All participants were recruited from the Department of Occupational and Environmental Medicine at the University Hospital in Örebro, Sweden. Approximately 50% of the participators had drunk coffee within an hour prior to exposure and one participant had used tobacco (snuff) prior to exposure. All the participants were right-handed, therefore, the measurement only was conducted on the right hand (Table 1).

**Table 1.** Descriptive statistics of study participants.

		Exposed cases		Unexposed controls	
		*N*	%	*N*	%
Gender	Male	6	67	3	50
	Female	3	33	3	50
Age	Mean	44		55	
	Median	41		57	
Nicotine use	Yes	1	11	0	0
	No	8	89	6	100
Dominant hand	Right	9	100	6	100
	Left	0	0	0	0

The average vibration exposure (calculated in cumulative daily A(8) value) was 4.1 m/s^2^ on the polishing tool’s right and left handle (Table 2).

**Table 2.** Descriptive statistics of hand-arm vibration dose for exposed subjects. The vibration exposure was measured with a triaxial accelerometer placed on both handles of the polishing tool.

	N	Mean	Median	Min	Max	SD^a^
Vibration dose; left hand (m/s^2^)^b^	9	4.1	5.0	2.1	5.0	1.3
Vibration dose; right hand (m/s^2^)	9	4.1	4.6	2.6	4.8	1.0

^a^Standard deviation.

^b^Meters per second squared.

Three of the samples taken from index fingers were discarded and could not be analyzed; two of them were taken before and one was taken after vibration exposure. Three of the samples taken from little fingers before the vibration exposure were discarded. These samples could not be analyzed because the blood had coagulated inside the collecting tubes.

The results from blood status analysis showed an increase of B-EVF, B-Hb, B-RBC, and B-WBC, as well as a decrease of PLT, ercMCV, ercMCH, and ercMCHC from index fingers after vibration exposure. Of these, only B-EVF was determined to be significantly different after exposure (*p* value = 0.04). In addition, there was a significant increase in B-neutrophils after vibration exposure (*p* value = 0.04; Table 3). For the unexposed controls, blood status analysis showed a decrease in Hb and EVF, but no changes were observed for ercMCV, ercMCH, ercMCHC, and B-WBC, including differential counting of lymphocytes, neutrophils, eosinophils, basophils, and monocytes.

**Table 3.** Capillary blood analyses from the index finger before and after hand-arm vibration exposure and at corresponding times for unexposed controls. The Wilcoxon signed-ranks test was used to determine *p* values. Statistically significant changes (*p* < 0.05) are indicated in bold. Number of samples omitted due to blood coagulation are also indicated.

Capillary sample*	Unexposed									Exposed							
	Before/After	Tested *N*	Omitted *N*	Mean	Median	Std	Min	Max	*p* value	Tested *N*	Omitted *N*	Mean	Median	Std	Min	Max	*p* value
B-EVF	Before	6	0	41.17	41.00	2.71	37.00	44.00	0.85	8	1	41.13	41.00	3.48	37.00	47.00	0.04
	After	6	0	40.50	39.50	4.18	36.00	48.00		7	2	42.86	44.00	3.19	39.00	47.00	
B-Hb	Before	6	0	140.17	142.00	12.17	124.00	165.00	0.34	8	1	145.25	142.50	13.38	128.00	170.00	0.18
	After	6	0	139.67	134.00	16.67	126.00	159.00		7	2	149.00	151.00	14.51	132.00	170.00	
B-RBC	Before	6	0	4.75	4.85	0.39	4.10	5.20	0.35	8	1	4.88	4.90	0.46	4.10	5.50	0.08
	After	6	0	4.75	4.70	0.58	4.10	5.70		7	2	5.07	5.10	0.38	4.50	5.50	
B-WBC	Before	6	0	5.47	5.50	0.80	4.20	6.60	0.72	8	1	5.10	5.10	0.97	3.60	7.00	0.14
	After	6	0	5.13	5.00	1.53	2.70	6.90		7	2	5.39	5.10	0.98	4.50	7.20	
B-PLT	Before	6	0	210.00	214.00	30.74	159.00	241.00	**0.04**	8	1	218.88	224.50	47.84	141.00	298.00	0.61
	After	5	1	177.60	181.00	30.14	136.00	217.00		7	2	203.86	212.00	47.59	147.00	276.00	
ercMCV	Before	6	0	86.00	86.50	3.74	82.00	90.00	1.00	8	1	84.88	84.50	2.85	80.00	90.00	0.08
	After	6	0	86.00	86.50	2.76	82.00	89.00		7	2	84.57	85.00	2.44	80.00	88.00	
ercMCH	Before	6	0	29.50	30.00	1.38	27.00	31.00	0.56	8	1	30.00	29.50	1.93	27.00	33.00	1.00
	After	6	0	29.67	30.00	1.86	26.00	31.00		6	3	29.33	29.50	1.37	27.00	31.00	
ercMCHC	Before	6	0	342.33	342.50	10.67	326.00	354.00	0.92	8	1	352.75	351.50	10.50	335.00	366.00	0.31
	After	6	0	342.67	350.00	13.56	322.00	257.00		7	2	348.14	349.00	10.21	332.00	359.00	
B-neutrophils	Before	6	0	2.82	2.80	0.73	1.90	3.80	1.00	8	1	2.69	2.50	0.64	1.70	3.90	**0.04**
	After	6	0	2.67	2.85	1.11	1.0	4.0		7	2	2.80	2.70	0.61	2.00	4.00	
B-lymphocytes	Before	6	0	1.90	1.90	0.28	1.60	2.30	0.89	8	1	1.71	1.75	0.38	1.10	2.10	0.59
	After	6	0	1.83	1.85	0.22	1.50	2.10		7	2	1.80	1.80	0.41	1.20	2.40	
B-monocytes	Before	6	0	0.47	0.50	0.10	0.30	0.60	0.18	8	1	0.41	0.40	0.12	0.20	0.60	0.41
	After	6	0	0.40	0.40	0.14	0.20	0.60		7	2	0.46	0.40	0.15	0.30	0.70	
B-eosinophils	Before	6	0	0.13	0.13	0.03	0.08	0.17	0.83	8	1	0.20	0.15	0.10	0.12	0.37	0.68
	After	6	0	0.13	0.13	0.05	0.04	0.18		7	2	0.21	0.15	0.08	0.13	0.34	
B-basophils	Before	6	0	0.05	0.05	0.02	0.02	0.08	0.32	8	1	0.05	0.05	0.02	0.02	0.09	0.71
	After	6	0	0.05	0.05	0.02	0.02	0.08		7	2	0.06	0.05	0.02	0.03	0.09	

*EVF: erythrocyte volume fraction, B-Hb: hemoglobin, B-RBC: red blood cell count, WBC: white blood cell count, PLT: platelet/thrombocyte count, ercMCV: mean cell volume, ercMCH: mean cell hemoglobin, ercMCHC: mean cell hemoglobin concentration, Std: Standard deviation.

For samples taken in the little finger, the results from blood status analysis showed an increase of B-EVF, B-Hb, B-RBC, B-WBC, PLT, and B-neutrophils after vibration exposure. There was no difference for ercMCV, ercMCH, and ercMCHC for the little finger after vibration exposure. For the differential counting of B-WBC, there was an increase in neutrophils, a decrease in basophils, and no changes for the other subgroups. No significant differences were found before and after vibration exposure regarding the samples that were taken from the little finger (see Supplement Table).

## Discussion

In this study, capillary blood samples before and after vibration exposure were compared. The study showed a significant increase in EVF and B-neutrophils in the index finger after vibration exposure. The increase of EVF can be compared with other studies that have shown an increase of EVF in venous blood samples, when compared before and after vibration exposure (Greenstein and Kester, 1997). The increase of EVF may be explained by the fact that vibration exposure leads to increased vascular permeability with subsequent plasma leakage which could lead to increased concentration of erythrocytes in the blood sample. This has also been shown in studies on mice (Okada et al., 1987).

A significant increase in B-neutrophils was also found. In a similar earlier study where blood samples were taken from the back of the hands, a tendency toward a decreased B-neutrophils count was shown, but the finding was not statistically significant (Greenstein and Kester, 1998). The differences between the studies may indicate that the transformation of the B-neutrophils takes place in the capillaries rather than in the veins, or that the dilution factor is so prominent that the increase of B-neutrophils cannot be shown in venous samples. Reduced blood flow and vasospasm suggests that the transformation takes place in the capillaries. It has been shown in earlier studies that an increase of leukotriene B4 occurs locally in VRP, which activates migration of leucocytes as well increases the production of free radicals. Thus, the increase of B-neutrophils in our study is supportive of that mechanism (Lau et al., 1992; Temprano, 2016). Activation of the leucocytes can also be a response to vascular endothelial damage, and together with the release of free radicals this could lead to further tissue damage (Stoyneva et al., 2003).

During the apoptosis of neutrophils, they release condensed chromatin and proteases called neutrophil extracellular traps (NETs). NETs form net structures of different forms, which have a vital function in bacterial defense, though it has also been shown that NETs have a prothrombotic effect and can adhere to erythrocytes and thrombocytes (Yang et al., 2016).

In this study, no significant changes could be observed among the hematological analyses or differential count of leucocytes other than B-EVF and B-neutrophils. A tendency toward increased B-Hb, B-RBC, and B-WBC, as well as in the differential count of leucocytes, were however noted. B-Hb in both the index and little fingers was increased after vibration exposure, which has also been shown in an earlier study (Stoyneva et al., 2003).

The present explanation of VRP is a combination of neural dysfunction, dysfunctional vasoregulation, and changes in blood viscosity (Stoyneva et al., 2003). The increase in erythrocytes and B-neutrophils that has been shown in acute vibration exposure can lead to increased viscosity and cause inflammatory processes, which could lead to tissue damage and further inflammatory responses. The tissue damages could in turn lead to neural dysfunction and alter the local vasoregulation(Kvietys and Granger, 2012). The present understanding of the pathophysiology of VRP conforms to the changes we observed in our study during acute vibrating exposure, and these changes could play a part in the development of VRP.

The study also included blood samples from unexposed controls. In this group, the platelet count was significantly lower at the second sampling instance. This might be a spurious result (Type-1 error), but could also indicate that platelets are consumed locally in the fingertip as a result of clot formation during the first lancet blood sampling (Locatelli et al., 2021; Yadegari et al., 2019). This remains a topic for further study.

Even though the sample size was small, we believe that the methodology was valid. The study was conducted under the same conditions for all participants, and the same personnel conducted the exposure measurement and blood sampling, which minimized the potential for procedural differences. Capillary samples that were collected during the study were taken on the opposite side of the index and little fingers before and after vibration exposure. This was done to avoid the potential for puncture damage made by the lancets effecting the results. A further strength is that the analyses were performed on capillary blood rather than venous blood, as has been used in earlier studies where a diluting factor from the affected capillary area can interfere with the result. This could also be an explanation of the different result when compared with other studies that have used venous blood. However, due to the small sample size in this study, the findings need to be confirmed in a larger study with similar methodology using capillary blood sample.

The main weakness of this study was that the study population was small. In addition, the results were hampered because of some samples having to be discarded before analysis due to coagulation in the sampling tubes. This probably occurred because of the rather large volume of capillary blood (250 μL) that had to be taken in a rather short period of time before analysis, and the sampling tubes remaining uncapped and unmixed for too long prior to analysis. The small population resulted in low statistical power, increasing the risk of false negative results, thus, further confirmatory investigations with a larger population is needed.

Furthermore, it cannot be ruled out that an inflammatory response was evoked as a result of the puncture damage that occurred, despite the fact that steps were taken to avoid this by conducting the blood sampling on different sides on the tip of the index and little fingers before and after vibration exposure. To account for this potential source of error, we also performed the same blood sampling procedure without vibration exposure to investigate the effect of the puncture damage, and in this group there were no effects on the neutrophils and EVF.

## Conclusion

This study indicated that acute vibration exposure to the hands among healthy subjects might induce a local increase of EVF and B-neutrophils measured in capillary blood taken from fingers. The increase of EVF and B-neutrophils that we observed in our study is in line with earlier studies on the mechanisms of the effect of vibration, with increased viscosity and inflammatory processes that cause tissue damage in the fingers.

Owing to the small population used in this study, further confirmatory investigations with a larger population and an improved method for collecting the blood samples are suggested.

## Supplemental Material

Supplemental Material - Effects on blood parameters from hand-arm vibrations exposure Click here for additional data file. Supplemental Material for Effects on blood parameters from hand-arm vibrations exposure by Niclas Johansson, Oscar Ragnebro, Albin Stjernbrandt, Pål Graff, Ing-Liss Bryngelsson and Per Vihlborg in Toxicology and Industrial Health.

## Appendix

### Abbreviations

EVF: erythrocyte volume fraction
ercMCH: mean cell hemoglobin
ercMCHC: mean cell hemoglobin concentration
ercMCV: mean cell volume
HAV: hand-arm vibration
HAVS: hand-arm vibration syndrome
Hb: hemoglobin
NETs: neutrophil extracellular traps
PLT: platelet/thrombocyte count
RBC: red blood cell
SD: standard deviation
VRP: vibration-induced Raynaud’s phenomenon
WBC: white blood cell

## References

[bibr1-07482337231173733] Bovenzi M (1986) Some pathophysiological aspects of vibration-induced white finger. European Journal of Applied Physiology and Occupational Physiology 55(4): 381–389. 3758038 10.1007/BF00422737

[bibr2-07482337231173733] Damon DH (1998) Postganglionic sympathetic neurons express endothelin. The American Journal of Physiology 274(3): R873–R878. 9530258 10.1152/ajpregu.1998.274.3.R873

[bibr3-07482337231173733] Eriksson K Burström L Nilsson T (2020) Blood biomarkers for vibration-induced white fingers. A case-comparison study. American Journal of Industrial Medicine 63(9): 779–786. 32597543 10.1002/ajim.23148

[bibr4-07482337231173733] Gemne G (1994) Pathophysiology of white fingers in workers using hand-held vibrating tools. Nagoya Journal of Medical Science 57(Suppl): 87–97. 7708114

[bibr5-07482337231173733] Greenstein D Kester RC (1997) The hemorheologic effects of hand-transmitted vibration. Angiology 48(9): 813–819. 9313631 10.1177/000331979704800908

[bibr6-07482337231173733] Greenstein D Kester RC (1998) The role of leukocytes in the pathogenesis of vibration-induced white finger. Angiology 49(11): 915–922. 9822048 10.1177/000331979804901107

[bibr7-07482337231173733] Heinonen E Färkkilä M Forsström J , et al. (1987) Autonomic neuropathy and vibration exposure in forestry workers. British Journal of Industrial Medicine 44(6): 412–416. 3606971 10.1136/oem.44.6.412 PMC1007843

[bibr50-07482337231173733] International Organization for Standardization (2001) Mechanical vibration—measurement and evaluation of human exposure to hand-transmitted vibration—Part 2: Practical guidance for measurement at the workplace. ISO 5349-2:2001.

[bibr8-07482337231173733] Kent PJ Williams GA Kester RC (1994) Platelet activation during hand vibration. The British Journal of Surgery 81(6): 815–818. 8044590 10.1002/bjs.1800810608

[bibr9-07482337231173733] Klevestedt A (2018) The Work Environment 2017 Volyme. 2. Stockholm, Sweden: Swedish Work Environment Authorithy.

[bibr10-07482337231173733] Kvietys PR Granger DN (2012) Role of reactive oxygen and nitrogen species in the vascular responses to inflammation. Free Radical Biology & Medicine 52(3): 556–592. 22154653 10.1016/j.freeradbiomed.2011.11.002 PMC3348846

[bibr11-07482337231173733] Lau CS O'Dowd A Belch JJ (1992) White blood cell activation in Raynaud’s phenomenon of systemic sclerosis and vibration induced white finger syndrome. Annals of the Rheumatic Diseases 51(2): 249–252. 1312815 10.1136/ard.51.2.249 PMC1005668

[bibr12-07482337231173733] Locatelli L Colciago A Castiglioni S , et al. (2021) Platelets in wound healing: what happens in space? Frontiers in Bioengineering and Biotechnology 9: 716184. 34760877 10.3389/fbioe.2021.716184 PMC8572965

[bibr13-07482337231173733] Nawaz I Nawaz Y Nawaz E , et al. (2022) Raynaud’s phenomenon: reviewing the pathophysiology and management strategies. Cureus 14(1): e21681. 35242466 10.7759/cureus.21681 PMC8884459

[bibr14-07482337231173733] Nilsson T Wahlström J Burström L (2017) Hand-arm vibration and the risk of vascular and neurological diseases-A systematic review and meta-analysis. PloS One 12(7): e0180795. 28704466 10.1371/journal.pone.0180795 PMC5509149

[bibr15-07482337231173733] Noël B (2000) Pathophysiology and classification of the vibration white finger. International Archives of Occupational and Environmental Health 73(3): 150–155. 10787129 10.1007/s004200050021

[bibr16-07482337231173733] Okada A Inaba R Furuno T , et al. (1987) Usefulness of blood parameters, especially viscosity, for the diagnosis and elucidation of pathogenic mechanisms of the hand-arm vibration syndrome. Scandinavian Journal of Work, Environment & Health 13(4): 358–362. DOI: 10.5271/sjweh.2027. 3433039

[bibr17-07482337231173733] Prete M Fatone MC Favoino E , et al. (2014) Raynaud’s phenomenon: from molecular pathogenesis to therapy. Autoimmunity Reviews 13(6): 655–667. 24418302 10.1016/j.autrev.2013.12.001

[bibr18-07482337231173733] Stoyneva Z Lyapina M Tzvetkov D , et al. (2003) Current pathophysiological views on vibration-induced Raynaud’s phenomenon. Cardiovascular Research 57(3): 615–624. 12618223 10.1016/s0008-6363(02)00728-9

[bibr19-07482337231173733] Temprano KK (2016) A review of Raynaud’s disease. Missouri Medicine 113(2): 123–126. 27311222 PMC6139949

[bibr20-07482337231173733] Yadegari M Pourabdian S Forouharmajd F (2019) Using the blood coagulation factors as a predictor component of the occupational vibration exposure. International Journal of Preventive Medicine 10: 150. 31579153 10.4103/ijpvm.IJPVM_337_17 PMC6767807

[bibr21-07482337231173733] Yang H Biermann MH Brauner JM , et al. (2016) New insights into neutrophil extracellular traps: mechanisms of formation and role in inflammation. Frontiers in Immunology 7: 302. 27570525 10.3389/fimmu.2016.00302 PMC4981595

