# Supplementary material for: Effects on blood parameters from hand-arm vibrations exposure
Source: Toxicol Ind Health. 2023 Apr 28;39(6):291–7. doi: 10.1177/07482337231173733 (PMC10210194; doi:10.1177/07482337231173733)
Supplement: Supplemental Material - Effects on blood parameters from hand-arm vibrations exposure [file sj-pdf-1-tih-10.1177_07482337231173733.pdf]

Supplement Table. Capillary blood analyses from the little finger before and after hand-arm vibration exposure. The Wilcoxon signed-ranks test was used to determine p values. Number of samples omitted due to blood coagulation are indicated.

				exposed					
Capillary sample	Before/ After	tested N	Omitted N	Mean	Median	Std	Min	Max	P-value
B-EVF	Before	6	3	40.83	40.50	2.79	38.00	46.00	0.33
	After	9	0	42.00	43.00	3.04	39.00	48.00	
B-Hb	Before	6	3	142.83	140.00	12.42	130.00	166.00	0.10
	After	9	0	146.78	147.00	11.60	130.00	171.00	
B-RBC	Before	6	3	4.78	4.80	0.38	4.40	5.40	0.11
	After	9	0	4.94	5.00	0.41	4.40	5.60	
B-WBC	Before	6	3	4.97	5.15	0.65	3.70	5.60	0.46
	After	9	0	5.23	5.20	0.82	4.10	6.80	
B-PLT	Before	6	3	205.33	207.50	51.04	138.00	283.00	0.92
	After	9	0	214.78	227.00	53.53	130.00	283.00	
ercMCV	Before	6	3	85.33	86.00	3.08	80.00	89.00	0.18
	After	9	0	85.22	84.00	3.53	79.00	91.00	
ercMCH	Before	6	3	29.83	30.00	1.84	27.00	32.00	0.56
	After	9	0	29.89	30.00	1.90	26.00	32.00	
ercMCHC	Before	6	3	349.67	353.50	12.14	334.00	361.00	0.35
	After	9	0	349.78	349.00	10.59	331.00	365.00	
B-neutrophils	Before	6	3	2.62	2.50	0.61	1.70	3.40	0.53
	After	9	0	2.83	2.80	0.56	1.80	3.90	
B-lymphocytes	Before	6	3	1.68	1.75	0.31	1.20	2.10	1.00
	After	9	0	1.63	1.80	0.34	1.10	2.00	
B-monocytes	Before	6	3	0.38	0.40	0.10	0.20	0.50	0.32
	After	9	0	0.46	0.50	0.11	0.30	0.60	
B-eosinophils	Before	6	3	0.19	0.17	0.09	0.12	0.36	0.75
	After	9	0	0.23	0.17	0.10	0.13	0.43	
B-basophils	Before	6	3	0.05	0.05	0.01	0.03	0.06	0.05
	After	9	0	0.05	0.04	0.02	0.02	0.09	

EVF: erythrocyte volume fraction, B-Hb: hemoglobin, B-RBC: red blood cell count, WBC: white blood cell count, PLT: platelet/thrombocyte count, ercMCV: mean cell volume, ercMCH: mean cell hemoglobin, ercMCHC: mean cell hemoglobin concentration, Std: Standard deviation.
